# The Mediated Effect of Sports Confidence on Competitive State Anxiety and Perceived Performance of Basketball Game

**DOI:** 10.3390/ijerph20010334

**Published:** 2022-12-26

**Authors:** Da-Ran Chun, Mi-Young Lee, Sang-Woo Kim, Eun-Young Cho, Byoung-Hee Lee

**Affiliations:** 1 Graduated School of Physical Therapy, Sahmyook University, Seoul 01795, Republic of Korea; 2 Department of Physical Therapy, Sahmyook University, Seoul 01795, Republic of Korea; 3 Virtual Rehabilitation Lab, Sahmyook University, Seoul 01795, Republic of Korea; 4 Institutional Research Center, Sahmyook University, Seoul 01795, Republic of Korea

**Keywords:** sport confidence, competitive state anxiety, perceived performance, basketball player

## Abstract

This study aimed to determine the mediating effect of sports confidence on competitive state anxiety and perceived performance basketball game. This study was conducted on 219 Korean basketball players, including 101 men and 118 women who were either high school students (42), university students (96), or professional basketball players (81). The Sources of Sport Confidence Questionnaire (SSCQ), Revised Competitive State Anxiety Inventory-2 (CSAI-2R), and the perceived performance questionnaire was used to measure sports confidence, competitive state anxiety, and perceived performance, respectively. The results showed that self-confidence (*β* = z 0.552, *p* < 0.001) and the coaches’ leadership (*β* = 0.552, *p* < 0.001) were found to be factors that influenced perceived performance. The perceived performance showed a positive correlation with all the sports confidence subscales and self-confidence of the competitive state anxiety subscales (*p* < 0.01). However, it showed a negative correlation with cognitive and somatic anxiety (*p* < 0.01). Sports confidence had a statistically significant mediating effect between somatic anxiety and perceived performance and a statistically significant mediating effect between self-confidence and perceived performance (*p* < 0.05). It appeared that sports confidence and competitive state anxiety had a statistically significant effect on perceived.

## 1. Introduction

Basketball is a team sport that requires individual and collective performance and mandates precision, strength, and endurance tasks [1]. It is a competitive sport that has more physical contact with another person than any other sport. As a result, injuries can often occur repeatedly; and is often inevitable in the athlete’s life; therefore, they must be able to respond positively and reliably in terms of physical and psychological aspects [2,3]. Among the causes of athletes not maximizing their ability in sports include various psychological factors, such as tension, stress, high arousal, anxiety, and lack of confidence. Of these, anxiety can be a main cause [4].

Competitive anxiety refers to feelings of tension, concern, and apprehension in a competitive situation, and it occurs when the context of the game or match is intimidating to the players. This pattern varies from person to person and is a temporary phenomenon [5]. Martens et al. (1990). provided a multidimensional explanation for sports anxiety [4]. Their multidimensional theory suggests that anxiety consists of cognitive and somatic subcomponents. Cognitive anxiety is defined as the mental component of anxiety and is caused by negative expectations about success or negative self-evaluation. Somatic anxiety refers to the physiological and affective elements of the anxiety experience that develop directly. A third subcomponent, as discussed by Martens et al. (1990). is the individual difference factor of self-confidence. This encompasses an athlete’s global perception of confidence. Although not originally proposed as a subcomponent of anxiety, Martens et al. [6]. included self-confidence in their study on the anxiety-performance relationship. In a multidimensional anxiety theory, cognitive anxiety is hypothesized to have a negative linear relationship with performance; somatic anxiety is hypothesized to have a quadratic (inverted U-shaped) relationship with performance, and self-confidence is hypothesized to have a positive linear relationship with performance [6].

Vealey [7] argued that it is necessary to conceptualize a unique self-confidence construct that fits into a model based on the interactional paradigm, sport-specificity, and the distinction between personality traits and states. Because the construct being conceptualized is not general self-confidence but sports-specific self-confidence, the term “sports confidence” should be used. Sports confidence is defined as the belief or degree of certainty that individuals possess about their ability to be successful in sports. This affects the athletes’ state of anxiety and performance [3,7].

In sports, perceived performance refers to states such as the athletes’ physical ability, technical-tactical level, motion performance capability, confidence, and game preparation. Athletes become aware of their performance level in the game, and this perception affects the formation of motivation and confidence. Therefore, the player’s performance can be seen as perceived performance formed through the actual performance and experience, and it improves players’ confidence, which has a great influence on the actual performance of the ones [8,9].

Previous studies [3,4,5,6,7] have shown the effect of sports confidence, self-efficacy, self-care, or competitive anxiety on performance. We assume that there is a mediating effect of sports confidence on the relationship between competitive state anxiety and performance. There is a suggestion that it is necessary to consider the influence of variables such as age and affiliation in the study and that there is a difference in the level of sports anxiety in athletes according to gender and the type of individual or team sports [10]. Therefore, the purpose of this study was to investigate the level of sports confidence, competitive state anxiety, and perceived performance of basketball players grouped according to sex, age, affiliation, and career. The second purpose was to investigate the correlation between sports confidence, competitive anxiety, and perceived performance. The third purpose was to investigate the mediating effect of sports confidence on the relationship between competitive state anxiety and perceived performance.

## 2. Materials and Methods

### 2.1. Subjects

A total of 219 Korean high school, university, and professional male and female basketball players participated in this study. We targeted each high school, university, and basketball team located in Seoul. The subjects were asked to complete the survey, and no specific conditions were presented, and individuals who wished to participate voluntarily participated. Four responses with insincere answers were excluded. Therefore, the total number of participants was 215. Of these, 42 were high school students, 96 were university students, and 77 were professional basketball players.

The present study was approved by the Sahmyook University Institutional Review Board (2-1040781-A-N-012020063HR), and it was registered (KCT0006812) in the Clinical Research Information Service of the Republic of Korea. The objective of the study and its requirements were explained to the subjects, and all participants provided their consent. This was in accordance with the ethical principles of the Declaration of Helsinki.

### 2.2. Experimental Proedure

Because of the COVID-19 pandemic, the survey was conducted from 20 September 2020, to 31 December 2020, through an online survey form provided by the NAVER online platform. Naver Form provides a link to an online questionnaire so that subjects can conduct a non-face-to-face questionnaire. Online surveys are anonymous. We explained the contents and purpose of the questionnaire directly to the coach at a high school located in Seoul, and asked the coach to randomly select students. The coach sent a link to the online questionnaire provided by Naver Form to the students via smartphone and e-mail. University students and athletes conducted the online survey in the same way.

The Revised Competitive State Anxiety Inventory-2 (CSAI-2R), the Source of Sport Confidence Questionnaire (SSCQ), and the perceived performance questionnaire were used to measure competitive state anxiety, sports confidence, and perceived performance in basketball players, respectively. The questionnaire consisted of 44 questions, including sex, age, affiliation, and career-related questions.

### 2.3. Assessment Tools and Data Collection

#### 2.3.1. Sports Confidence

Sports confidence was measured using the Korea version of the SSCQ by Kim [11]. It consists of 15 questions; the subscales are demonstration of ability (three items), coaches’ leadership (four items), physical and mental preparation (four items), and social support (four items) and were analyzed using a confirmatory factor analysis (CFA) on the existing nine subscales by Vealey et al. [12]. The participants responded by circling a number of on a 7-point Likert scale ranging from 1 (not at all important) to 7 (of highest importance). High scores indicate a high level of sports confidence.

The results of Cronbach’s α was 0.90 for demonstration of ability, 0.77 for coaches’ leadership, 0.75 for physical and mental preparation, and 0.84 for social support [13].

#### 2.3.2. Competitive State Anxiety

The CSAI-2R was used to assess competitive state anxiety. It consists of 17 questions; the subscales are somatic anxiety (seven items), cognitive anxiety (five items), and self-confidence (five items). The respondents rated their feelings before a competition on a scale from 1 (not at all) to 4 (very much so). The subscale scores were calculated by summing the items in each subscale, dividing by the number of items, and multiplying by 10. The score ranges from 10 to 40 for each subscale [14]. The scores for cognitive and somatic anxiety indicate that higher scores indicate higher anxiety, and high self-confidence scores indicate increased confidence in performance.

The factorial validity of the CSAI-2R was previously established by Cox et al. [15]. using CFA on data from 331 athletes, which showed a good fit of the data on the model (comparative fit index [CFI] = 0.95, non-normed fit index [NNFI] = 0.94, root mean square error of approximation [RMSEA] = 0.05) [15]. In addition, the results of Cronbach’s *α* showed that it was 0.75 for cognitive anxiety, 0.85 for somatic anxiety, and 0.83 for self-confidence [14].

#### 2.3.3. Perceived Performance

Perceived performance was measured using the perceived performance questionnaire by Mamassis and Doganis [16]. It was developed to assess the perceived performance of tennis. The questionnaire consists of eight questions, each of which has a subscale. The participants responded to the questions on a 5-point scale, ranging from 1 (not good at all) to 5 (very good) on the following aspects: (1) their physical feelings, (2) quality of technique, (3) timing and rhythm, (4) concentration, (5) amount of effort exerted, (6) mental attitude and thoughts, (7) level of self-confidence during the match, and (8) comparison of their performance with what they were expected to play, given the opponent. High scores indicate high performance [16].

The reliability of the eight items was 0.84. The results of CFA, CFI, Tucker–Lewis index (TLI), and goodness of fit index (GFI) were above 0.80–0.90, and RMSEA was below 0.06 [17].

#### 2.3.4. Data Analysis

All the general characteristics of the subjects were normally distributed. SPSS statistical software (IBM, Chicago, IL, USA, 2013) version 22.0 was used for all statistical analyses. Differences in variables according to the general characteristics were analyzed using the *t*-test and ANOVA, and results were presented as mean ± standard deviation. The Least Significant Difference (LSD) test was used for the post hoc test. Correlation and multiple regression analyses were used to examine the correlations between variables.

To examine whether sports confidence has a mediating effect on competitive state anxiety and perceived performance relationships, it was divided into cognitive anxiety, somatic anxiety, and self-confidence, which are subscales of competitive state anxiety. The SPSS PROCESS macro program was used for the mediating effects [18]. The procedure for verification of the mediating effect was used as an analysis method by Barron and Kenny [19]. Step 1: The independent variable should have a significant effect on the mediator variable (path *a*). Step 2, the mediator variable must have a significant effect on the dependent variable (path *b*). Step 3: The independent variable should have a significant effect on the dependent total effect (path *c*). Step 4: If the mediator variable adds path c, which is the direct effect (path *c’*), the effect of the independent variable (*β* value) should be reduced. If the effect of the independent variable (*β* value) is not significant, it is called complete mediation; and if the effect of the independent variable (*β* value) is significant, it is called partial mediation [19]. The bootstrap confidence interval was used to verify that the indirect effect of the mediation model was statistically significant. For all tests, the level of statistical significance was set at *p* < 0.05.

## 3. Results

Table 1 shows the general characteristics of the participants, which included sex, age, affiliation, and career.

### 3.1. Comparison of Sports Confidence

The results of the difference in sports confidence according to sex, age, affiliation, and career are shown in Table 2. The mean scores for demonstration of ability were 17.57 in men and 16.98 in women. The mean scores for coaches’ leadership were 22.49 in men and 20.92 in women (*p* < 0.05). The mean scores for physical and mental preparation were 23.70 in men and 23.25 in women. The mean score for social support were 24.07 in men and 23.61 in women. Differences in sports confidence according to sex showed significant differences only in coaches’ leadership.

The mean scores for demonstration of ability were 17.35 for those between ages 10–19, 17.19 for those in between ages 20–29, and 17.39 for those between ages 30–39 years. The mean scores for coaches’ leadership were 22.58 for those between ages 10–19, 21.19 for those in between ages 20–29, and 22.39 for those between ages 30–39 years. The mean scores for physical and mental preparation were 23.14 for those between ages 10–19, 23.23 for those between ages 20–29, and 25.11 for those between ages 30–39 (*p* < 0.05). In the post hoc analysis, players between ages 30–39 had a higher mean score for physical and mental preparation for the game than those between ages 10–19 and 20–29 years. The mean scores for social support were 24.53 for those between ages 10–19, 23.50 for those between ages 20–29, and 24.36 for those between ages 30–39 years. Differences in sports confidence based on age showed significant differences only in physical and mental preparation.

The results of the mean scores for demonstration of ability were 17.50 in high school, 17.08 in university, and 17.31 in professional players. The mean scores for coaches’ leadership were 22.67 in high school, 21.54 in university, and 21.17 in professional players. The mean scores for physical and mental preparation were 23.38 in high school, 22.96 in university, and 24.12 in professional players. The mean scores for social support scores were 24.55 in high school, 23.75 in university, and 23.51 in professional players. There were no significant differences in sports confidence according to affiliation.

The results of the mean scores for demonstration of ability were 16.94 for within 5 years, 17.47 for within 10 years, 17.24 for within 20 years, and 16.33 for over 20 years of experience. The mean scores for coaches’ leadership were 21.50 for within 5 years, 22.19 for within 10 years, 20.53 for within 20 years, and 22.13 for over 20 years. The mean scores for physical and mental preparation were 22.59 for within 5 years, 23.45 for within 10 years, 23.40 for within 20 years, and 25.67 for over 20 years. The mean scores for social support were 23.47, 23.96, 23.84, and 23.47 for 5, 10, 20, and for over 20 years, respectively. There were no significant differences in sports confidence based on career.

### 3.2. Comparison of Competitive State Anxiety

The results of the difference in competitive state anxiety according to sex, age, affiliation, and career are shown in Table 3. The mean scores for cognitive anxiety were 21.26 in men and 24.34 in women (*p* < 0.05). The mean scores for somatic anxiety were 17.86 in men and 20.31 in women (*p* < 0.05). The mean scores for self-confidence were 29.13 in men and 24.42 in women (*p* < 0.05). Differences in competitive state anxiety according to sex showed a significant difference in all subscales between men and women.

The results of the mean score for cognitive anxiety were 24.70 in between the ages 10–19, 22.88 for those between the ages 20–29, and 20.64 for those between the ages 30–39 (*p* < 0.05). In the post hoc analysis, players between the ages 10–19 had more cognitive anxiety in the game than players in the 30–39 age group. The mean somatic anxiety scores were 19.10 in those aged 10–19, 19.76 for those aged 20–29, and 16.53 for those aged 30–39 years (*p* < 0.05). In the post hoc analysis, the players in the 20–29 age group had more somatic anxiety in the game than those in 30–39 age group. The mean scores for self-confidence were 25.81 in those ages 10–19, 26.21 for those ages 20–29, and 29.43 for those ages 30–39 (*p* < 0.05). In the post hoc analysis, players in the 30–39 age group had more self-confidence in the game than those in the 10–19 and 20–29 age groups. Differences in competitive state anxiety according to age showed a significant difference in all the subscales between the ages of 10–19, 20–29, and 30–39 years.

The mean scores for cognitive anxiety were 24.48 in high school, 23.35 in university, and 21.61 in professional basketball players(*p* < 0.05). In the post hoc analysis, high school players had more cognitive anxiety in the game than professional players. The mean scores for somatic anxiety were 18.91 in high school, 19.90 in university, and 18.52 in professional basketball players. The mean scores for self-confidence were 25.86 in high school, 25.92 in university, and 27.71 in professional basketball players. Differences in competitive state anxiety according to affiliation showed significant differences only in cognitive anxiety between high school, university, and professional basketball players. There were no significant differences in somatic anxiety or self-confidence.

The results of the mean scores for cognitive anxiety were 22.88 for within 5 years, 23.81 for within 10 years, 22.41 for within 20 years, and 18.93 for those who were in the career for over 20 years (*p* < 0.05). In the post hoc analysis, who had within 5 years of career experience had more cognitive anxiety in the game than players with over 20 years of career experience. The mean scores for somatic anxiety were 20.34 for within 5 years, 19.63 for within 10 years, 18.55 for within 20 years, and 16.19 for over 20 years. The mean scores for self-confidence were 26.0 for within 5 years, 25.85 for within 10 years, 26.79 for within 20 years, and 31.87 for over 20 years (*p* < 0.05). In the post hoc analysis, players with over 20 years of career experience showed higher self-confidence in the game than other players. Differences in competitive state anxiety according to career experience length showed significant differences in cognitive anxiety and self-confidence.

### 3.3. Comparison of Perceived Performance

The results of the difference in perceived performance according to sex, age, affiliation, and years within the career are shown in Table 4. The mean scores for perceived performance were 29.94 in men and 27.18 in women (*p* < 0.05). Differences in perceived performance according sex showed a significant difference in all subscales between men and women.

For the difference in competitive state anxiety according to age, the mean scores for perceived performance were 27.44 in the 10–19, 28.60 in the 20–29, and 29.00 in the 30–39 age categories. There were no significant differences between groups.

For the difference in competitive state anxiety according to affiliation, the mean scores for perceived performance were 27.67 in high school, 28.43 in university, and 28.83 in professional basketball players. There were no significant differences between groups.

The mean scores for perceived performance were 28.09, 28.19, 28.76 for within 5, 10, 20 years, respectively, and 29.53 for over 20 years of career experience. There were no significant differences in competitive state anxiety based on career experience length.

### 3.4. Correlation Analysis

The correlations between sports confidence, competitive state anxiety, and perceived performance are shown in Table 5. The demonstration of ability was positively correlated with coaches’ leadership (*r* = 0.648), physical and mental preparation (*r* = 0.700), social support (*r* = 0.662), self-confidence (*r* = 0.284), and perceived performance (*r* = 0.341). Coaches’ leadership showed a positive correlation with physical and mental preparation (*r* = 0.661), social support (*r* = 0.678), self-confidence (*r* = 0.385), and perceived performance (*r* = 0.465), whereas it was negatively correlated with somatic anxiety (*r* = −0.134). Physical and mental preparation showed a positive correlation with social support (*r* = 0.706), self-confidence (*r* = 0.452), and perceived performance (*r* = 0.483), whereas it was negatively correlated with cognitive (*r* = −0.231) and somatic anxiety (*r* = −0.267). Social support was positively correlated with self-confidence (*r* = 0.364) and perceived performance (*r* = 0.434). Cognitive anxiety was positively correlated with somatic anxiety (*r* = 0.703), whereas it was negatively correlated with self-confidence (*r* = −0.484) and perceived performance (*r* = −0.352). Somatic anxiety showed a negative correlation with self-confidence (*r* = −0.363) and perceived performance (*r* = −0.225). Self-confidence was positively correlated with perceived performance (*r* = 0.650).

### 3.5. Multiple Regression Analysis

Multiple regression analysis was conducted to determine the impact of sports confidence and competitive state anxiety on perceived performance. The Durbin-Watson test was used to determine the autocorrelation coefficient of the dependent variable. As a result, the Durbin-Watson index was 2.250, and the dependent variable was independent, regardless of autocorrelation. The regression model showed an explanatory power of 47.1% on perceived performance, self-confidence (*β* = 0.552, *p* < 0.001), and coaches’ leadership (*β* = 0.253, *p* < 0.001), which were factors that influenced perceived performance (Table 6).

### 3.6. Analyses of Mediating Effects

#### 3.6.1. Analysis of the Mediating Effect of Sports Confidence on the Relationship between Somatic Anxiety and Perceived Performance of Basketball Players

**Figure 1:**
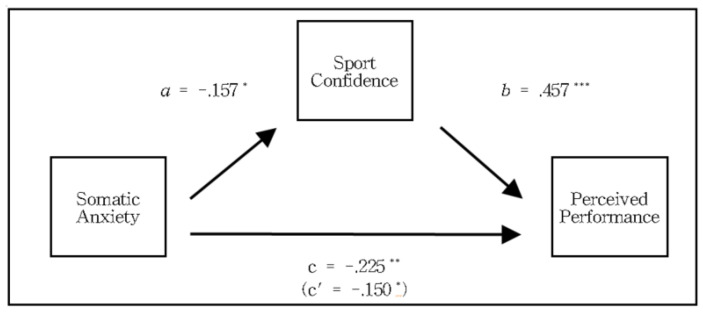
Mediating effect of sports confidence on the relationship between somatic anxiety and perceived performance. * *p* < 0.05, ** *p* < 0.01, *** *p* < 0.001.

The results of the mediating effect of sports confidence on the relationship between somatic anxiety and perceived performance of basketball players are shown in Table 7. The effect of somatic anxiety, which is an independent variable, on sports confidence as a mediator variable (path a) was statistically significant (*β* = −0.157, *t* = −2.326, *p* < 0.05), and *R*^2^ was 0.051; the effect of sports confidence, which is a mediating variable on perceived performance as a dependent variable (path b) was statistically significant (*β* = 0.457, *t* = 8.003, *p* < 0.01). The direct effect of somatic anxiety on perceived performance (path c’) was also statistically significant (*β* = −0.150, *t* = −2.528, *p* < 0.05). Therefore, the effect of basketball players’ somatic anxiety on perceived performance by mediating sports confidence is statistically significant. The direct effect of somatic anxiety on perceived performance is also significant, which supports the partial mediation model (Figure 1).

The bootstrap confidence interval was used to verify that the mediating effect of the mediation model was statistically significant. Verification showed that the mediating effect (indirect effect) was statistically significant because it did not contain 0 within the confidence interval (low limit confidence interval [LLCI] = −0.112, upper limit confidence interval [ULCI] = −0.012) which was estimated to be −0.075. The significance of the mediating effect (indirect effect) was determined by Sobel *Z* verification, and the indirect effect was significant at *Z* = −2.218 (*p* < 0.05). Therefore, somatic anxiety affects the perceived performance of basketball players, but somatic anxiety also affects perceived performance through sports confidence.

#### 3.6.2. Analysis of the Mediating Effect of Sports Confidence on the Relationship between Self-Confidence and Perceived Performance of Basketball Players

**Figure 2:**
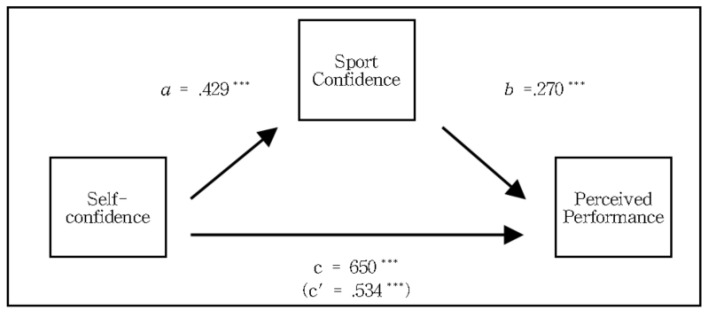
Mediating effect of sports confidence on the relationship between somatic anxiety and perceived performance. *** *p* < 0.001.

The results of the mediating effect of sports confidence on the relationship between self-confidence and perceived performance of basketball players are shown in Table 8. The effect of self-confidence, which is an independent variable, on sports confidence as a mediating variable (path a), was statistically significant (*β* = −0.429, *t* = 6.939, *p* < 0.01), and the effect of sports confidence, which is a mediating variable on perceived performance as a dependent variable (path b), was statistically significant (*β* = 0.270, *t* = 4.923, *p* < 0.01). In addition, direct effect of self-confidence on perceived performance (path c’) was statistically significant (*β* = 0.534 *t* = 9.747, *p* < 0.01). Therefore, the effect of basketball players’ self-confidence on perceived performance by mediating sports confidence is statistically significant. The direct effect of self-confidence on perceived performance is also significant, which supports the partial mediation model (Figure 2).

The bootstrap confidence interval was used to verify that the mediating effect of the mediation model was statistically significant. Verification showed that the mediating effect (indirect effect) was statistically significant because it did not contain 0 within the confidence interval (LLCI = 0.050, ULCI = 0.124), which was estimated to be 0.116. The significance of the mediating effect (indirect effect) was determined by Sobel *Z* verification, and the indirect effect was significant at *Z* = 3.988 (*p* < 0.001). Therefore, self-confidence affects the perceived performance of basketball players, but self-confidence also affects perceived performance through sports confidence.

## 4. Discussion

In this study, cognitive and somatic anxiety was high in women, and self-confidence and perceived performance were high in men, as in other studies [20,21,22]. In terms of sports confidence, it was high in men for the coaches’ leadership subscale (*p* < 0.01). In terms of age, hose in the 10–19 age category had the lowest levels of cognitive, somatic anxiety, and self-confidence, and those in the age 30–39 age range had the highest levels of cognitive, somatic anxiety, and self-confidence. In terms of sports confidence, physical and mental preparation was high for those in their thirties. In terms of career length, those with more than 20 years of experience showed the lowest cognitive anxiety and highest self-confidence (*p* < 0.05).

As shown above, women experienced more anxiety than men, and self-confidence in the game was higher in men. Schaal et al. [21] reported that genetic, physiological, and socio-environmental factors can lead female athletes to be more psychologically unstable and sensitive to the difficulties they encounter in the environment. Therefore, it is believed that female athletes show less confidence than male athletes because of their high levels of anxiety. Additionally, it was found that athletes with more than 20 years of career experience had lower cognitive anxiety and higher self-confidence. This is believed to help veteran players control their anxiety and increase their confidence in the process of overcoming difficulties. To take advantage of confidence during performance, it would be better to educate athletes on the concept and necessity of sports confidence by dividing them according to affiliation and length of career experience.

In this study, all of the sports confidence subscales had a positive correlation with perceived performance (*p* < 0.01). Sports confidence was positively correlated with performance in competitive athletes who completed state measures of confidence level/sources, imagery type and performance within one hour after competition [23]. Levy et al. [23] reported that coaches’ leadership, demonstration of ability, and physical and mental preparation, which are subscales of sports confidence, have been shown to correlate with performance. Internal and external parts, such as the subscales of sports confidence, directly affect the performance of athletes. A decrease in any of them is likely to have a significant impact on their performance. It seems to have a positive impact on performance as people support the athletes and they experience psychological stability by believing in themselves. Thus, high sports confidence can facilitate the athlete’s perceived performance.

In this study, coaches’ leadership had a negative correlation with somatic anxiety (*p* < 0.05), and physical and mental preparation had a negative correlation with cognitive and somatic anxiety (*p* < 0.01). Bum [24] reported that coaches’ leadership had a negative correlation with cognitive anxiety in golf players. Lim et al. [25] reported that coaches’ leadership had a negative correlation with somatic anxiety, and physical and mental preparation had a negative correlation with cognitive and somatic anxiety in university male golfers. In women’s basketball, only the main players participated in the game; therefore, there are not many opportunities for various players to prove their abilities. Leaders often pressurize players rather than encourage them; thus, players seem to rely on themselves more than on the people around them.

In this study, cognitive and somatic anxiety had a negative correlation with perceived performance, and self-confidence showed a positive correlation with perceived performance (*p* < 0.01). Bum and Shin [26] reported that cognitive anxiety had a significant negative correlation with performance, and self-confidence had a significant positive correlation with performance in golfers. Chamberlain and Hale [27] found a negative relationship between cognitive anxiety and the athletes’ performance in sports. Self-confidence was positively correlated with cognitive performance and performance skill. It seems that the players’ performance was negatively affected by cognitive and somatic anxiety and was positively affected by high self-confidence. Perceived performance reflects an inner commitment to successfully demonstrate skills or performance [28]. A number of factors affect performance, including genetics, fitness level, training status, training period, psychological state at the time of the game, and the external environment of the stadium [24,26]. From the above research results, it is inferred that performance in sports events can be determined by changes in physical, mental, technical aspects and individual factors of the players.

As a result of the proportional relationship between performance and anxiety, confidence and anxiety had a negative impact on all players. However, Burton [29] and Swain et al. [30] reported that the tension and concentration caused by slight anxiety had a positive effect on competition when the extreme anxiety factor was removed. If the cause of anxiety could be identified by each position, such as starting, bench, and individual players, and if one could present a way to relieve anxiety so that the players could recognize the positive side of anxiety, they would be able to improve their performance through this understanding.

In this study, self-confidence and coaches’ leadership were found to be factors that influenced perceived performance (*p <* 0.01). As in our studies, some studies [31,32] reported that sports confidence significantly influenced performance in athletes. Players tend to rely on their leaders because the outcome of the game is determined by the leaders’ decisions during the game. It seems that it can have a positive impact on performance by accepting the leader’s operation, performing it confidently, and showing good performance. Therefore, the coach, who maintains the closest relationship to the players, should have the players adapt to competitive situations through practice training and should set small goals during training to give them a sense of accomplishment and increase self-confidence.

Sports confidence is an important driving force for improving athletes’ performance [33]. When athletes have confidence in their sports performance, it can successfully lead to good performance; but when they lack confidence, their athletic performance can be poor [20]. Kim [34] reported that sports confidence had a mediating effect (partial mediation) between the athletes’ belief on their ability and perceived performance in judo players. Besharat and Pourbohlool [20] reported that self-confidence had a moderating effect (partial) between competitive anxiety and sport performance. Levy et al.’s study explored the mediating role of sport confidence upon (1) sources of sport confidence-performance relationship and (2) imagery-performance relationship [23]. Although previous studies have not observed the mediating effect of sports confidence on the relationship between competitive state anxiety and perceived performance, sports confidence was shown to have a mediating effect on different relationships. This study also showed that sports confidence can improve the negative relationship between competitive state anxiety and perceived performance. Sports confidence had a statistically significant mediating effect on somatic anxiety and perceived performance (*p* < 0.05). Sport confidence had a statistically significant mediating effect between self-confidence and perceived performance (*p* < 0.05). Sports confidence helps athletes manage anxiety better and contribute to results.

As with some literature [35,36], it is believed that a program that can improve the sub-factors of sports confidence that have a mediating effect can have a positive impact on performance if it educates the athletes, trainers, and the people around them. The limitation of the study was that the questionnaire was not conducted immediately before or after the competition, and the difference in general characteristics did not appear clearly. It was difficult to generalize the results because of the failure to conduct a complete enumeration survey of Korean basketball players. In addition, it was not considered that there could be differences in levels of confidence, anxiety, and perceived performance of the main and non-main players, even if they belong to the same team.

## 5. Conclusions

According to the study, the more experienced the players, the lower the anxiety and the higher the self-confidence. Given that sports confidence has a mediating effect between competitive state anxiety and perceived performance, athletes with anxiety before the start of the game may be able to increase their performance with sports confidence. Players with a strong belief in their ability reported to be able to peak under pressure and cope successfully with adverse situations during competition [33], similarly, it was confirmed in our study. To do so, support from family, coaches, and trainers are required. Moreover, players should praise and encourage themselves and actively prepare their bodies and minds to show their abilities in the field. It would be better to create programs that could improve confidence for separate groups, such as the main players, non-main players, and individual tendencies. As confirmed in our study, since sports confidence, anxiety, and performance differ according to sex and age, it is necessary to construct differentiated programs according to the level of the group. In addition, it would be good to inform players about the positive aspects of anxiety to their lower cognitive and somatic anxiety so that they could adapt to anxiety in their training. Since there is a lack of research on anxiety and performance of basketball players, which are representative competitive sports, and the mediating effects of sports confidence, it is hoped that this study will continue to study competitive state anxiety, performance, and sports confidence.

## Figures and Tables

**Table 1:** General characteristics of participants (*n* = 215).

Parameters		Participation	%
Sex	Male	97	45.1
	Female	118	54.9
Age (yrs)	10–19	43	20.0
	20–29	144	67.0
	30–39	28	13.0
Affiliation	High school	42	19.5
	University	96	44.7
	Professional	77	35.8
Career	Within 5 years	34	15.8
	6–10 years	108	50.2
	11–20 years	58	27.0
	Over 20 years	15	7.0

**Table 2:** Comparison of sports confidence (*n* = 215).

Parameters		DA	CL	PMP	SS
Sex	Male	17.57 ± 3.46	22.49 ± 4.02	23.70 ± 3.88	24.07 ± 4.13
	Female	16.98 ± 3.45	20.92 ± 4.56	23.25 ± 3.72	23.61 ± 3.79
	t(p)	1.233 (0.219)	2.664 (0.008)	0.859 (0.391)	0.853 (0.394)
Age	10–19 (A)	17.35 ± 3.26	22.58 ± 3.56	23.14 ± 3.89	24.53 ± 3.14
	20–29 (B)	17.19 ± 3.59	21.19 ± 4.73	23.23 ± 3.89	23.50 ± 4.22
	30–39 (C)	17.39 ± 3.16	22.39 ± 3.38	25.11 ± 2.67	24.36 ± 3.48
	*F(p)*	0.064 (0.938)	2.168 (0.117)	3.124 (0.046)	1.443 (0.239)
	*Post-hoc*			A, B *<* C	
Affiliation	High school	17.50 ± 2.97	22.67 ± 3.48	23.38 ± 3.47	24.55 ± 3.18
	University	17.08 ± 3.73	21.54 ± 4.61	22.96 ± 4.20	23.75 ± 4.27
	Professional	17.31 ± 3.38	21.17 ± 4.51	24.12 ± 3.34	23.51 ± 3.91
	*F(p)*	0.232 (0.793)	1.627 (0.199)	2.025 (0.135)	0.971 (0.380)
Career	Within 5 years	16.94 ± 3.38	21.50 ± 3.86	22.59 ± 3.98	23.47 ± 3.99
	6–10 years	17.47 ± 3.62	22.19 ± 4.37	23.45 ± 4.02	23.96 ± 4.21
	11–20 years	17.24 ± 3.25	20.53 ± 4.78	23.40 ± 3.29	23.84 ± 3.45
	Over 20 years	16.33 ± 3.31	22.13 ± 3.56	25.67 ± 2.80	23.47 ± 4.00
	*F(p)*	0.587 (0.624)	1.881 (0.134)	2.342 (0.074)	0.175 (0.913)

Values are expressed as mean ± standard deviation; DA, demonstration of ability; CL, coaches’ leadership; PMP, physical and mental preparation; SS, social support.

**Table 3:** Comparison of competitive state anxiety (*n* = 215).

Parameters		CA	SA	SC
Sex	Male	21.26 ± 5.95 ^a^	17.86 ± 4.38	29.13 ± 5.97
	Female	24.34 ± 6.63	20.31 ± 6.37	24.42 ± 5.78
	*t(p)*	−3.550 (0.000)	−3.330 (0.001)	5.857 (0.000)
Age	10–19 (A)	24.70 ± 6.35	19.10 ± 5.24	25.81 ± 5.36
	20–29 (B)	22.88 ± 6.62	19.76 ± 5.86	26.21 ± 6.53
	30–39 (C)	20.64 ± 5.47	16.53 ± 4.72	29.43 ± 5.88
	*F(p)*	3.400 (0.035)	3.906 (0.022)	3.906 (0.032)
	*Post-hoc*	C ˂ A	C ˂ B	A, B ˂ C
Affiliation	High school (A)	24.48 ± 6.26	18.91 ± 5.14	25.86 ± 5.42
	University (B)	23.35 ± 6.63	19.90 ± 5.84	25.92 ± 6.45
	Professional (C)	21.61 ± 6.30	18.52 ± 5.73	27.71 ± 6.49
	*F(p)*	3.304 (0.050)	1.337 (0.265)	2.069 (0.129)
	*Post-hoc*	C ˂ A		
Career	Within 5 years (A’)	22.88 ± 6.19	20.34 ± 5.12	26.00 ± 5.12
	6–10 years (B’)	23.81 ± 6.72	19.63 ± 5.89	25.85 ± 6.80
	11–20 years (C’)	22.41 ± 6.05	18.55 ± 5.38	26.79 ± 5.51
	Over 20 years (D’)	18.93 ± 6.09	16.19 ± 5.68	31.87 ± 5.93
	*F(p)*	2.743 (0.044)	2.363 (0.072)	4.298 (0.006)
	*Post-hoc*	D’ ˂ A’		A’, B’, C’ ˂ D’

Values are expressed as mean ± standard deviation; CA, cognitive anxiety; SA, somatic anxiety; SC, self-confidence.

**Table 4:** Comparison of perceived performance (*n* = 215).

Parameters		Perceived Performance
Sex	Male	29.94 ± 4.57
	Female	27.18 ± 4.07
	t(p)	4.682 (0.000)
Age	10–19	27.44 ± 4.61
	20–29	28.60 ± 4.44
	30–39	29.00 ± 4.67
	*F(p)*	1.370 (0.256)
Affiliation	High school	27.67 ± 4.55
	University	28.43 ± 4.62
	Professional	28.83 ± 4.35
	*F(p)*	0.907 (0.405)
Career	Within 5 years	28.09 ± 4.50
	6–10 years	28.19 ± 4.58
	11–20 years	28.76 ± 4.33
	Over 20 years	29.53 ± 4.85
	*F(p)*	0.562 (0.640)

Values are expressed as mean ± standard deviation.

**Table 5:** Correlation analysis (*n* = 215).

Parameters	DA	CL	PMP	SS	Cog	Som	Scon	Per
DA	1							
CL	0.648 **	1						
PMP	0.700 **	0.661 **	1					
SS	0.662 **	0.678 **	0.706 **	1				
Cog	−0.004	−0.088	−0.231 **	−0.106	1			
Som	−0.031	−0.134 *	−0.267 **	−0.107	0.703 **	1		
Scon	0.284 **	0.385 **	0.452 **	0.364^**^	−0.484 **	−0.363 **	1	
Per	0.341 **	0.465 **	0.483 **	0.434^**^	−0.352 **	−0.225 **	0.650 **	1

DA, demonstration of ability; CL, coaches’ leadership; PMP, physical and mental preparation; SS, social support; Cog, cognitive anxiety; Som, somatic anxiety; Scon, self-confidence; Per, perceived performance. * *p* < 0.05, ** *p* < 0.01.

**Table 6:** Multiple regression analysis (*n* = 215).

Dependent Variable (Perceived Performance)		*B*	*SE*	*β*	*t*	*R* ^2^	Adj *R^2^*	*F*
Model 1	(constant)	16.102	1.015		15.856 ***	0.422	0.419	155.511 ***
	Self-confidence	0.464	0.037	0.650	12.470 ***			
Model 2	(constant)	12.332	1.258		9.799 ***	0.476	0.471	96.444 ***
	Self-confidence	0.395	0.038	0.552	10.257 ***			
	Coaches’ leadership	0.260	0.055	0.253	4.693 ***			

Except demonstration of ability, cognitive anxiety, somatic anxiety, physical and mental preparation, and social support; Durbin-Watson = 2.250. *** *p* < 0.001.

**Table 7:** Analysis of the mediating effect of sports confidence on the relationship between somatic anxiety and perceived performance (*n* = 215).

Dependent Variable (Perceived Performance)		*B*	*SE*	*β*	*t*	LLCI	ULCI
	Somatic anxiety (*c*) ^b^	−0.178	0.053	−0.225	−3.369 **	−0.283	−0.074
1 step	*R(R* ^2^ *)*	0.225 (0.051)					
	*F*	11.353 **					
Dependent variable (Perceived performance)		*B*	*SE*	*β*	*t*	LLCI	ULCI
	Somatic anxiety (*a*) ^b^	−0.376	0.162	−0.157	−2.326 *	−0.694	−0.057
2 step	*R(R* ^2^ *)*	0.157 (0.025)					
	*F*	5.412 *					
Dependent variable (Perceived performance)		*B*	*SE*	*β*	*t*	LLCI	ULCI
	Sports confidence (*b*) ^b^	0.158	0.020	0.457	8.003 ***	0.119	0.197
3 step	Somatic anxiety (*c’*)	−0.119	0.047	−0.150	−2.528 *	−0.212	−0.026
	*R(R* ^2^ *)*	0.520 (0.271)					
	*F*	39.378 ***					
Sobel Z							
		*B*	*β*	Boot *S.E*	Boot LLCI	Boot ULCI
Bootstrap		−0.059	−0.075	0.025	−0.112	−0.012
*Z*				−2.218 *		

LLCI, lower limit confidence interval; ULCI, upper limit confidence interval; ^b^, Path. * *p* < 0.05, ** *p* < 0.01, *** *p* < 0.001.

**Table 8:** Analysis of the mediating effect of sports confidence on the relationship between self-confidence and perceived performance (*n* = 215).

Dependent Variable (Perceived Performance)		*B*	*SE*	*β*	*t*	LLCI	ULCI
	Self-confidence (*c*) ^b^	0.464	0.037	0.650	12.470 ***	0.391	0.537
1 step	*R(R* ^2^ *)*	0.650 (0.422)					
	*F*	155.511 ***					
Dependent variable (Perceived performance)		*B*	*SE*	*β*	*t*	LLCI	ULCI
	Self-confidence (*a*) ^b^	0.923	0.133	0.429	6.939	0.661	1.185
2 step	*R(R* ^2^ *)*	0.429 (0.184)					
	*F*	48.154 ***					
Dependent variable (Perceived performance)		*B*	*SE*	*β*	*t*	LLCI	ULCI
	Sports confidence (*b*) ^b^	0.090	0.018	0.270	4.923 ***	0.054	0.125
3 step	Self-confidence (*c’*)	0.381	0.039	0.534	9.747 ***	0.304	0.459
	*R(R* ^2^ *)*	0.694 (0.481)					
	*F*	98.359 ***					
Sobel Z							
		*B*	*β*	Boot *S.E*	Boot LLCI	Boot ULCI
Bootstrap		0.083	0.116	0.019	0.050	0.124
*Z*				3.988 ***		

LLCI, low limit confidence interval; ULCI, upper limit confidence interval; ^b^, Path. *** *p* < 0.001.

## Data Availability

Not applicable.
